# Early Sialadenitis After Radioactive Iodine Therapy for Differentiated Thyroid Cancer: Prevalence and Predictors

**DOI:** 10.1155/2020/8649794

**Published:** 2020-08-04

**Authors:** Ruba Riachy, Nisrine Ghazal, Mohamad B. Haidar, Ahmad Elamine, Mona P. Nasrallah

**Affiliations:** ^1^Department of Internal Medicine, Division of Endocrinology and Metabolism, American University of Beirut Medical Center, Beirut, Lebanon; ^2^Department of Endocrinology, University of Texas Medical Branch, Galveston, TX, USA; ^3^Department of Endocrinology, Dr. Sulaiman Al Habib-Medical Group, Dubai, UAE; ^4^Department of Radiology, Division of Nuclear Medicine, American University of Beirut Medical Center, Beirut, Lebanon

## Abstract

**Introduction:**

Sialadenitis is a frequent occurrence after radioactive iodine therapy (RAI). However, reports on its predictors and risk factors in the Eastern Mediterranean Region (EMRO) are scarce.

**Aim:**

This study aimed to identify risk factors for early sialadenitis in patients receiving RAI for differentiated thyroid cancer (DTC) at the American University of Beirut Medical Center. It also aimed to determine the prevalence and characteristics of such patients receiving RAI at our institution.

**Methods:**

This was a retrospective study conducted at the American University of Beirut Medical Center. Medical charts were reviewed for all patients 18–79 years of age admitted to receive RAI for DTC between 01/01/2012 and 31/12/2015. Sialadenitis was deemed present if there were any records of neck swelling/pain, dry mouth, or difficulty swallowing within 48 hours of RAI administration. Characteristics between patients with sialadenitis and those without were compared to determine predictors.

**Results:**

There were 174 patients admitted to receive RAI, predominantly females (71.3%), with papillary thyroid cancer (93.1%). The majority had lymph node involvement (64.5%). Pretreatment thyroid stimulating hormone (TSH) was greater than 75 mIU/ml in most patients (72.6%). The prevalence of sialadenitis was 20.1% (95% CI (15–27)). Being non-Lebanese and having a positive whole-body scan were associated with sialadenitis and persisted after adjustments (OR = 2.34 and 3.99). Non-Lebanese patients had higher rates of lymph nodes involvement (*p* value 0.005) and were kept off levothyroxine for longer periods (*p* value 0.02).

**Conclusion:**

The prevalence of sialadenitis at our institution was similar to other reported studies from the world. However, risk factors allude to more iodine exposure in the neck with positive whole-body scan uptake, lymph node involvement, and prolonged period of hypothyroidism.

## 1. Introduction

Radioactive iodine (RAI) has been the core therapy in treating multiple thyroid diseases. It was first used to treat Grave's disease in 1942 [1] and differentiated thyroid cancer (DTC) in 1946 [2]. In DTC, RAI can eradicate normal thyroid remnants and neoplastic microfoci and therefore facilitate follow-up of the patients' serum thyroglobulin levels and iodine whole-body scans [3, 4]. In clinical practice, especially prior to the introduction of recombinant TSH use, a TSH level of 30 mIU/ml or above is aimed at before giving RAI to allow for optimal remnant thyroid uptake and ablation. However, unforeseen effects secondary to RAI therapy have been identified, namely, salivary gland inflammation (sialadenitis), the risk of secondary malignancies, hematological abnormalities, conjunctivitis, and alopecia [5]. Among those, sialadenitis is the most frequent complication encountered, with a wide world prevalence estimate varying from 2% to 67% in different studies [6].

Physiologic uptake after RAI is expected in the liver, stomach, oropharynx, nasopharynx, esophagus, and salivary glands [7]. The parotid, submandibular, and sublingual glands have been reported to concentrate iodine as high as 7–700 times the plasma levels [8]. Salivary gland dysfunction occurs when iodine accumulates and substitutes for chloride as a substrate in the sodium-potassium-chloride (Na/K/Cl) cotransporter, which can, therefore, result in sialadenitis, xerostomia, and taste disturbances [9]. Iodide can also be transported directly into the salivary glands, which express the sodium-iodide symporter or NIS [10]. Sialadenitis can appear in the early phase of RAI administration secondary to salivary gland inflammation (within 7 days of treatment) or later after 7 days [11]. Indeed, in a review paper by Mandel and Mandel, salivary gland swelling and pain, namely, of the parotid gland, were the main reported symptoms after RAI treatment. In addition, secondary complications including xerostomia, taste alterations, and increases in caries were reported [12]. These symptoms developed immediately or months later and tended to progress in intensity over time.

Risk factors to develop sialadenitis are linked to the radioactive iodine uptake and include age, gender, RAI dose, and preexisting salivary gland disease [13]. However, studies in the literature that tackle sialadenitis predictors are limited. Given the fact that the condition can be bothersome to patients, it would be important to identify potentially unforeseen risk factors, which may be reversible. Furthermore, no such studies exist on the prevalence and predictors of sialadenitis in the Eastern Mediterranean Region (EMRO).

## 2. Objectives

This study aimed to determine the prevalence and risk factors for early (i.e., within 48 hours) sialadenitis in patients receiving RAI in Lebanon, a middle-income country of EMRO.

## 3. Methods

### 3.1. Patients

The study was conducted at the American University of Beirut Medical Center (AUBMC), which is a major referral center to the Lebanese population, as well as citizens of other neighboring countries from the EMRO region, especially Syria and Iraq. Twelve centers provide RAI in Lebanon, out of which seven are active. The load seen at AUBMC constitutes about 18–24% of the country load, which allows for a good reflection on the population studied.

### 3.2. Inclusion/Exclusion Criteria

All consecutive patients between 18 and 79 years, admitted to AUBMC to receive RAI for DTC, were included. Patients receiving RAI for hyperthyroidism (toxic nodule, toxic multinodular goiter, and Grave's disease) and those with salivary gland pathology (stone, tumor, and surgery) were excluded.

### 3.3. Data Collection

This was a retrospective study conducted through medical charts' revision of patients admitted to receive RAI between January 1, 2012, and December 31, 2015. These patients were admitted for 48 hours in a special isolation room. The following variables were recorded: date of RAI, date of birth, age, gender, nationality, weight, height, Body Mass Index (BMI), blood pressure, tumor pathology, recurrence, lymph node involvement, RAI dose, days off levothyroxine (LT4)/days after thyroidectomy, smoking status, alcohol intake, known dental problems, recombinant TSH (thyrogen) intake, pretreatment TSH level, and post-RAI whole-body scan (WBS) results. Recurrence was considered present if the patient had documented prior treatment with RAI. In addition, data was extracted from WBS performed one week after RAI treatment to detect the presence of remnant tissue after ablation. WBS results were reported as negative (no uptake), positive (uptake in the neck), and distant (uptake in the neck and at distant sites). If sialadenitis (as defined below) was present, treatment of sialadenitis was also recorded into the data collection sheet.

Two of the authors conducted data extraction. To ensure homogeneity in data collection and in defining sialadenitis, the other investigator blindly filled ten charts for each investigator, and the concordance rate in detecting sialadenitis and extracting the information in the chart was almost 100%.

### 3.4. Diagnosis of Sialadenitis

In the majority of cases, the authors relied on notes and assessments by endocrinology fellows on call for the diagnosis of sialadenitis, detailing the clinical assessment of patients with symptoms of sialadenitis, including neck swelling and pain. In the absence of a clear assessment note, sialadenitis was deemed present if there were any records of neck swelling, neck pain, dry mouth, or difficulty swallowing within 48 hours of RAI administration. In addition, whether subjects required treatment with nonsteroidal anti-inflammatory drugs or glucocorticoids was noted.

### 3.5. Statistical Analysis

Continuous variables were reported as mean ± (SD), while categorical variables were reported as number (percent). Univariate analysis was performed by independent *t*-test for continuous variables and chi square test for categorical variables. Since nationality was found to be a possible risk factor to develop sialadenitis, the same analysis was repeated with nationality stratification (Lebanese versus non-Lebanese).

Multivariate analysis of potentially significant predictors of sialadenitis was also performed. Predictors included gender, age, lymph node involvement, RAI dose, days off LT4, and WBS result. When confounders were suspected, the analysis was undertaken again with isolation of one of these variables. *p* value <0.05 was used to indicate significance. Analyses were done using SPSS version 23.

### 3.6. Ethical Considerations

AUBMC Institutional Review Board approved the study.

## 4. Results

Based on the retrospective review of medical records, the total number of patients admitted to receive RAI from January 2012 to December 2015 was 230. Out of these, 56 patients received iodine for benign thyroid pathology and were therefore excluded. The remaining 174 patients had all undergone total thyroidectomy followed by RAI for histologically confirmed DTC.

### 4.1. Patients' Characteristics

Patients admitted for RAI for DTC at our institution had a mean age of 42.8 ± 14.4 years and were predominantly women (71.3%). The patients were mostly Lebanese, but around one-third were from other nationalities, mainly Iraqi and Syrian. The majority had papillary thyroid cancer (93.1%), while follicular cancer accounted for 5.7% only. The DTC was recurrent in 14.9% and 64.5% of the patients had lymph node involvement. Most patients received 100 millicuries (mCi) (63.2%), followed by 50 mCi (23%), while 13.8% received 150 mCi (Table 1). The average of mCi received per year was 90.3 mCi for 2012, 93.7 mCi for 2013, 97.6 mCi for 2014, and 102.8 mCi for 2015. Only 4% received recombinant TSH prior to RAI. Almost all the patients (96%) relied on LT4 withdrawal, and the majority (72.6%) had a pretreatment TSH greater than 75 mIU/ml. The mean duration for RAI after thyroidectomy off any replacement was 42.4± days while the mean for RAI after LT4 withdrawal was 26.8± days (Table 1). There were 47 patients who did not have available WBS results. Out of the remaining 127 subjects, 77.2% had positive neck uptake by WBS 7–10 days after RAI, while 7.9% had distant uptake and 15% had no uptake (Table 1).

### 4.2. Prevalence of Sialadenitis

Of the 174 patients, 35 (20.1%) [95% CI (15–27)] had sialadenitis within the first 48 hours of iodine intake. Out of these, 17 (48.6%) received nonsteroidal anti-inflammatory drugs (NSAIDs) for symptomatic management, 3 (8.6%) were treated with steroids, 6 (17.1%) got dual therapy (NSAIDs and steroids), and 9 (25.7%) were not treated pharmacologically (Table 1).

### 4.3. Risk Factors for Sialadenitis

The association between sialadenitis and the studied parameters is outlined in Table 1.

There were twice as many non-Lebanese subjects who developed salivary gland dysfunction (30.4%), as there were Lebanese (15.3%), with a *p* value of 0.02. Age, gender, tumor pathology, recurrence, and lymph node involvement were not statistically significant when comparing patients with sialadenitis to patients without. Sialadenitis rates were not different across RAI doses, nor by recombinant TSH intake, LT4 withdrawal duration, or WBS results.

Because of the significant association with nationality, the analysis was stratified by nationality and the abovementioned risk factors were studied (Table 2). Non-Lebanese patients receiving RAI for DTC had higher rates of lymph node involvement (*p* value of 0.005) and were kept off LT4 for longer periods (mean 37.1 vs. 20.6 days, *p* value 0.02). They also had a higher BMI and systolic and diastolic blood pressure.

Independent risk factors for sialadenitis, using multivariate analysis as outlined in Table 3, were the following: nationality persisted as a risk factor with non-Lebanese having an odds ratio (OR) of 2.34 [95% CI (1.07–5.13)] to develop sialadenitis as compared to Lebanese patients. In this analysis, a positive WBS was also a risk factor for sialadenitis development with ORs of 3.99 [95% CI (1.13–14.16)]. We further repeated the analysis removing nationality and lymph nodes involvement one at a time. Both positive WBS and nationality remained significant (data not shown). Finally, due to large number of patients with unknown WBS results, a sensitivity analysis was done without WBS and nationality persisted as significant predictor (data not shown).

## 5. Discussion

Patients admitted to receive RAI at our institution were predominantly women with PTC, and the majority had lymph node involvement. The prevalence of sialadenitis was 20.1% and its independent risk factors were being non-Lebanese and having a positive postiodine WBS. In turn, subjects who were from other nationalities had more lymph node involvement and were kept off LT4 for longer times.

TSH level per se did not clearly stand out as a predictor for sialadenitis. However, TSH levels in our study were found to be higher than the recommended level of 30 mIU/mL or above.

The reported prevalence rate of sialadenitis is highly variable in the literature. For instance, An et al. found that symptomatic late onset sialadenitis occurred at a rate of 10.2% [14] while Lee et al. reported 46.3% rate of chronic sialadenitis based on subjective symptoms [13]. Hyer et al. showed that 26% of patients developed salivary gland toxicity months after RAI therapy, and 15% developed the symptoms in the first 48 hours [11]. Alexander et al. reported the prevalence of acute and chronic sialadenitis as 33.0% and 42.9%, respectively [5]. The high variability may relate to the method used to diagnose sialadenitis and to the timing (early versus late). Nonetheless, our reported rate falls within the range of early sialadenitis described in the literature.

Concerning risk factors, although sialadenitis might not be very common with lower, often diagnostic, doses of RAI [15], the literature generally reports the dose of RAI administered as a predictor for sialadenitis. Lee et al. found that RAI dose was the only factor with a positive correlation to sialadenitis [13]. A study by Hollingsworth et al. reported RAI dose, older age, female gender, and a history of prior sialadenitis as risk factors for salivary gland dysfunction after treatment [16]. In addition, Hoffman et al. propose being more conservative with RAI or avoiding it when feasible in cases of low risk thyroid cancer to counteract the dose-dependent risk of sialadenitis [17]. Another study conducted by Iakovou et al. showed that the use of thyrogen for preparation of RAI ablation as opposed to LT4 withdrawal reduces the prevalence of salivary gland dysfunction, possibly due to reduced hypothyroidism [18]. In our study, we could not replicate the abovementioned factors, mainly because very few subjects received thyrogen. The majority of our patients took high doses of RAI (100 mCi). This factor may be reflective of the older guidelines for practice before RAI therapy use became more conservative [19]. However, it may also be reflective of AUBMC being a referral center with 30% of patients coming from outside the country possibly with more disease burden. Even though nationality was independently associated with sialadenitis, it is likely to be a confounder than an actual predictor. Given that risk factors found in non-Lebanese were lymph node involvement and longer LT4 withdrawal time, it is possible that the non-Lebanese in the study had more tumor load in the neck area and were also kept hypothyroid for a longer period, so that nationality was a surrogate marker for the former two risk factors. Therefore, the discrepancy is unlikely to be ethnic and more likely reflects a referral bias as well as logistical and travel constraints. The prolonged period of hypothyroidism may hypothetically act as a stimulant to the sodium-iodide symporter, thus increasing the amount of radioactivity in the salivary glands. Furthermore, the higher uptake in WBS, coupled to longer days off LT4, may decrease the transit time of RAI, increasing the exposure time of salivary glands to radioactivity [20]. The majority of the patients in our study received high doses of RAI, between 100 and 150 mCu compared with the 75 mCu found to substantially increase the risk of dry mouth and 125 mCu found to substantially increase the risk of sialadenitis [21], this may have limited our ability to demonstrate a correlation between administered radioiodine activity and sialadenitis activity, as a ceiling effect may be at play.

The novelty in the current study is that it targets early sialadenitis predictors, and no such reports have been conducted in the Eastern Mediterranean Region to our knowledge. Most of our patients had pretreatment TSH above 75 mIU/ml, way above the needed target to achieve adequate iodine uptake. The latter fact indicates that most patients are rendered symptomatically hypothyroid for longer than necessary. In fact, a goal TSH of equal or above 30 mIU/L is generally adopted in preparation for RAI therapy or diagnostic testing as per the 2015 American Thyroid Association (ATA) guidelines for thyroid nodules and DTC [19]. Furthermore, a study conducted by Serhal et al. showed that target level of TSH was achieved 18 days after thyroidectomy and 22 days after LT4 withdrawal in more than 95% of patients included in the study. This result encourages limiting the waiting period off thyroid hormone in patients awaiting the RAI treatment [22]. Another consideration would be recombinant TSH preparation as opposed to LT4 withdrawal. As per our results, recombinant TSH seems to be underutilized and would constitute a practical alternative when appropriate, sparing patient's undesirable symptoms while awaiting ablative treatment.

Finally, sialadenitis rates are considerable and the burden of the symptoms affect patients' quality of life; hence one can identify high-risk patients, look for early signs and symptoms and implement when appropriate preventive measures. The 2008 European Association of Nuclear Medicine (EANM) guideline for ^131^I therapy recommends sufficient hydration with use of lemon candy, sour candy, or chewing gum in the 24 h following ^131^I administration to increase salivary flow and to reduce radiation exposure of the salivary glands [23]. Patient education, hydration, withdrawal of anticholinergic medications, and noninflammatory and cholinergic medications administration are all potential preventive methods to consider [6].

Our study has the following limitations: first, being retrospective by design, data was collected from medical records and medication orders. Mild symptoms could have been neglected and/or not mentioned, and therefore sialadenitis may have been underdiagnosed.

In addition, the diagnosis of sialadenitis was based on subjective reports by the patients. Some symptoms of sialadenitis, such as neck pain, might have alternative etiologies. One example is higher concentrations of radioiodine in the thyroid of patients with relatively large residual thyroid tissue increase the risk of neck pain after treatment, in support of remnant tissue thyroiditis [24]. Conventional sialography and salivary scintigraphy are generally used to evaluate salivary gland dysfunction, and more recent data on less invasive magnetic resonance sialography is emerging [25]. Posttherapeutic ^131^I scintigraphy can provide important information on the risk of symptomatic sialadenitis in DTC with an 80–93% predictive value [26]. Nonetheless, while recording subjective symptoms would certainly be lacking in terms of sensitivity in comparison to objective measures, it would be of more clinical relevance and so far has been used in the majority of studies assessing risk factors either alone [16] or in addition to sialography [13, 14]. In addition, subjective symptoms do correlate well with the radiologic measures of sialadenitis. For example, Choi et al. demonstrated that MR sialography images were useful for evaluating RAI sialadenitis, and its findings were in accordance with disease severity [25]. Another study which tackled the clinical to radiologic association concluded that the number of dysfunctional salivary glands detected on scintigraphy was correlated with xerostomia (*p* < 0.01) [27].

In addition, this study only assessed early sialadenitis and results would not apply to the long-term effects. Although early sialadenitis may be transient in nature, it is still considered bothersome by many patients and can persist in some cases causing chronic symptoms.

Finally, it is worth noting that though dosimetry would be very useful to estimate RAI doses to the salivary glands, at our institution, it is not included in the RAI therapy protocol. As a consequence of the retrospective design of our study, it was not possible to implement it beforehand, and we were consequently unable to assess exact doses to the salivary glands and further elucidate the RAI dose-dependence of the risk of sialadenitis, as previously discussed.

## 6. Conclusion

Around 20% of patients admitted to receive RAI for DTC develop sialadenitis at our institution, which is comparable to rates reported in the literature. Having a positive posttreatment whole-body scan, more lymph nodes involvement, and longer LT4 withdrawal time are risk factors for salivary glands dysfunction. Therefore, we emphasize on giving special attention to patients with more aggressive disease. We also recommend avoiding rendering patients excessively hypothyroid as measures to prevent early development of sialadenitis.

## Figures and Tables

**Table 1:** Characteristics of subjects with and without sialadenitis who received radioactive iodine for differentiated thyroid cancer.

	Total	Sialadenitis		*p* value
	*N* = 174	No *N* = 139	Yes *N* = 35	
Gender *n* (%) female	124 (71.3)	96 (69.1)	28 (80.0)	0.20
Age, mean (±SD)	42.8 ± 14.4	43.0 ± 14.8	41.8 ± 13.1	0.65
Nationality *n* (%)				
Lebanese	118 (67.8)	100 (71.9)	18 (51.4)	0.02
Non-Lebanese	56 (32.2)	39 (28.1)	17 (48.6)
BMI, mean (±SD)	28.6 ± 6.3	28.4 ± 6.4	29.2 ± 5.7	0.49
SBP, mean (±SD)	125.1 ± 15.8	125.3 ± 15.9	124.4 ± 15.6	0.76
DBP, mean (±SD)	79.4 ± 11.7	79.5 ± 11.8	79.2 ± 11.6	0.92
Tumor pathology				
PTC	162 (93.1)	130 (93.5)	32 (91.4)	0.57
FTC	10 (5.7)	7 (5.0)	3 (8.6)	
Other	2 (1.1)	2 (1.4)	0 (0.0)
Recurrence, yes *n* (%)	26 (14.9)	23 (16.5)	3 (8.6)	0.24
Lymph node, yes *n* (%)	89 (64.5)	69 (62.7)	20 (71.4)	0.39
RAI dose *n* (%)				
50 m/Cu	40 (23.0)	36 (25.9)	4 (11.4)
100 m/Cu	110 (63.2)	84 (60.4)	26 (74.3)	0.18
150 m/Cu	24 (13.8)	19 (13.7)	5 (14.3)
Thyrogen, yes	7 (4.0)	6 (4.3)	1 (2.9)	1.00
Pretherapy TSH (mIU/L), mean (±SD)	83.9 ± 23.4	85.8 ± 21.7	76.6 ± 28.4	0.04
Pretherapy TSH (mIU/L) categorical^*∗*^				
<50	18 (11.0)	12 (9.2)	6 (17.6)	0.11
50–75	27 (16.5)	19 (14.6)	8 (23.5)
>75	119 (72.6)	99 (76.2)	20 (58.8)
Smoking, yes	39 (22.5)	31 (22.3)	8 (23.5)	0.88
Alcohol, yes	23 (13.3)	19 (13.7)	4 (11.8)	1.00
Dental problems, yes	3 (2.2)	3 (2.8)	0 (0.0)	1.00
Days off LT4, mean (±SD)	26.8 ± 19.4	24.9 ± 16.3	33.0 ± 27.9	0.63
Days after surgery, mean (±SD)	42.4 ± 38.3	44.3 ± 42.0	35.1 ± 15.9	0.12
Days off total, mean (±SD)	39.7 ± 36.2	41.0 ± 39.5	34.7 ± 18.5	0.18
WBS result^*∗∗*^				
Negative	19 (15.0)	17 (17.5)	2 (6.7)	0.16
Positive	98 (77.1)	71 (73.3)	27 (90.0)	
Distant	10 (7.9)	9 (9.2)	1 (3.3)
Treatment received				
NSAIDs	20 (11.5)	3 (2.2)	17 (48.6)	0.19
Steroids	5 (2.9)	2 (1.4)	3 (8.6)
Both	6 (3.4)	0 (0.0)	6 (17.1)

^*∗*^10 patients did not have TSH levels available; ^*∗∗*^47 patients did not have WBS results available.

**Table 2:** Characteristics of subjects who received radioactive iodine for differentiated thyroid cancer, stratified by nationality as Lebanese versus non-Lebanese.

	Nationality		*p* value
	Lebanese *N* = 118	Non-Lebanese *N* = 56	
Gender *n* (%)			
Male	31 (26.3)	19 (33.9)	0.30
Female	87 (73.7)	37 (66.1)
Age, mean (±SD)	44.2 ± 15.2	39.8 ± 12.2	0.06
BMI, mean (±SD)	27.4 ± 5.2	30.9 ± 7.6	0.004
SBP, mean (±SD)	123.5 ± 15.3	128.6 ± 16.4	0.05
DBP, mean (±SD)	78.1 ± 11.5	82.2 ± 11.8	0.03
Tumor pathology *n* (%)			
PTC	108 (91.5)	54 (96.4)	0.42
FTC	8 (6.8)	2 (3.6)	
Other	2 (1.7)	0 (0.0)
Recurrence, yes *n* (%)	17 (14.4)	9 (16.1)	0.77
Lymph node, yes *n* (%)	54 (56.8)	35 (81.4)	0.005
RAI dose *n* (%)			
50 mCi	30 (25.4)	10 (17.9)	0.38
100 mCi	74 (62.7)	36 (64.3)	
150 mCi	14 (11.9)	10 (17.9)
Thyrogen, yes	4 (3.4)	3 (5.5)	0.68
Pretherapy TSH (mIU/L) mean (±SD)	85.0 ± 22.2	81.5 ± 25.9	0.39
Pretherapy TSH (mIU/L)–categorical^*∗*^			
<50	9 (8.2)	9 (16.7)	0.25
50–75	18 (16.4)	9 (16.7)
>75	83 (75.5)	36 (66.7)
Smoking, yes *n* (%)	32 (27.4)	7 (12.5)	0.03
Alcohol, yes *n* (%)	21 (17.8)	2 (3.6)	0.01
Dental problems, yes *n* (%)	2 (2.1)	1 (2.3)	1.00
Days off LT4, mean (±SD)	20.6 ± 16.1	37.1 ± 20.8	0.02
Days after surgery, mean (±SD)	40.3 ± 28.1	47.5 ± 56.3	0.45
Days off total, mean (±SD)	37.2 ± 27.5	45.2 ± 50.7	0.19
WBS result^*∗∗*^*n* (%)			
Negative	12 (13.0)	7 (20.0)	0.31
Positive	71 (77.2)	27 (77.1)	
Distant	9 (9.8)	1 (2.9)
Treatment received *n* (%)			
NSAIDs	11 (9.3)	9 (16.1)	0.60
Steroids	3 (2.5)	2 (3.6)	
Both	2 (1.7)	4 (8.9)

^*∗*^10 patients did not have TSH levels available; ^*∗∗*^47 patients did not have WBS results available.

**Table 3:** Independent predictors for sialadenitis.

Sialadenitis (reference: no)		
Variables	OR (95% CI)	*p* value
Gender	2.34 (0.92–5.97)	0.07
Nationality	2.34 (1.07–5.13)	0.03
WBS positive	3.99 (1.13–14.16)	0.03

Variables included in the model were gender (reference: male); age; nationality (reference: Lebanese); lymph node involvement (reference: no); RAI dose (mCi) (reference: 50 mCi); days off LT4; and WBS result (reference: negative).

## Data Availability

The data generated in the study are included in this article. The database is available upon request.
